# Distinct Labels Attenuate 15-Month-Olds’ Attention to Shape in an Inductive Inference Task

**DOI:** 10.3389/fpsyg.2012.00586

**Published:** 2013-01-02

**Authors:** Susan A. Graham, Jean Keates, Ena Vukatana, Melanie Khu

**Affiliations:** ^1^Department of Psychology, University of CalgaryCalgary, AB, Canada; ^2^Department of Clinical Health Psychology, Faculty of Medicine, University of ManitobaWinnipeg, MB, Canada

**Keywords:** inductive inferences, categorization, word learning

## Abstract

We examined the role of distinct labels on infants’ inductive inferences. Thirty-six 15-month-old infants were presented with target objects that possessed a non-obvious property, followed by test objects that varied in shape similarity relative to the target. Infants were tested in one of two groups, a *Same Label* group in which target and test objects were labeled with the same noun, and a *Distinct Label* group in which target and test objects were labeled with different nouns. When target and test objects were labeled with the same count noun, infants generalized the non-obvious property to both test objects, regardless of similarity to the target. In contrast, labeling the target and test objects with different count nouns attenuated infants’ generalization of the non-obvious property to both high and low-similarity test objects. Our results suggest that by 15 months, infants recognize that object labels provide information about underlying object kind and appreciate that distinct labels are used to designate members of different categories.

## Introduction

Infants’ early categorization abilities permit them to organize the vast diversity of entities in their environment into categories, comprised of like kinds, and to use these categories as the basis for inductive inferences. That is, once a child categorizes an individual object as a member of a particular kind (e.g., a dog), she can make inferences about properties of that object (e.g., barks). This is a critical skill because many object properties are not immediately obvious in any particular encounter, and must be inferred in some way (e.g., dogs are loyal companions). Research has documented that naming plays an instrumental role in young children’s categorization and inferences: naming a set of objects with the same name highlights similarities among them that might otherwise have been overlooked on the basis of perceptual analysis alone (e.g., Gelman and Markman, 1986, 1987; Gelman and Coley, 1990; Welder and Graham, 2001). In the present study, we explored the role of distinct labels on infants’ inductive inferences. Specifically, we examined whether 15-month-old infants will infer that objects belong to different categories when they are labeled with different names, even if this label information conflicts with other cues to category membership (i.e., object similarity).

A large body of research has demonstrated that the sensitivity to linguistic cues for object categorization emerges early in development (e.g., Waxman and Markow, 1995; Waxman and Booth, 2001; Dewar and Xu, 2007; Plunkett et al., 2008). For example, words will facilitate category formation in infants as young as 3 months of age (Ferry et al., 2010). Importantly, this facilitation is specific to words; other types of auditory stimuli, such as tones and melodic sequences, do not guide categorization, even when paired consistently with objects (Fulkerson and Haaf, 2003, 2006; Fulkerson and Waxman, 2007; Ferry et al., 2010). Furthermore, naming only supports categorization when objects are labeled with the same name (vs. variable names; Waxman and Braun, 2005). Over the first 2 years of life, the facilitative influence of words becomes even more specified, focusing on the distinct role of nouns versus words from other form classes (i.e., adjectives; Waxman and Booth, 2001; Booth and Waxman, 2009).

The powerful and early emerging role of naming in categorization is also evident in inductive inference tasks (e.g., Gelman and Markman, 1986, 1987; Gelman and Coley, 1990; Booth and Waxman, 2002; Jaswal and Markman, 2007; Noles and Gelman, 2012). In these tasks, children are asked to determine whether test objects share the same non-obvious properties (i.e., properties that are not continuously available in the perceptual array) as a target object. The reasoning underlying these tasks is as follows: children will generalize the non-obvious property to those test objects that they view as members of the same category as the target. Studies have shown that when the target and test objects are introduced with the same count noun, 13- to 22-month-old infants assume the objects also share a non-obvious property, even when those objects share only minimal perceptual similarity (Welder and Graham, 2001; Graham et al., 2004; Graham and Kilbreath, 2007). In contrast, when objects are not labeled, infants assume that only objects that are highly similar in shape share non-obvious properties (Graham et al., 2004; Graham and Diesendruck, 2010).

Despite the strong consensus that words play a critical role in guiding categorization and inductive inferences, there is considerable debate around the underlying mechanisms and processes that account for the facilitative effect of words (e.g., Gelman and Waxman, 2007; Sloutsky et al., 2007). On one account, knowledge-based models follow the premise that an understanding of *kind* drives children’s inductive inferences (Xu et al., 1999; Carey, 2000; Mandler, 2000; Waxman and Gelman, 2009). Names are hypothesized to provide a marker of an object’s kind, such that objects that share the same name are more likely to share common properties (Gelman, 2003). However, it is important to note that knowledge-based models do not deny the importance of perceptual features; rather, perceptual features are said to be indicative of “deeper” similarities between objects. In contrast, proponents of similarity-based models argue that complex cognitive processes, such as inductive reasoning, are a result of powerful learning mechanisms (i.e., similarity assessment, attentional weighting, and associative learning; e.g., Smith et al., 1996; Sloutsky, 2003; Sloutsky and Fisher, 2004; Sloutsky et al., 2007). According to this view, linguistic labels act as an additional feature, which increase the overall similarity between two entities (Best et al., 2011; Deng and Sloutsky, 2011; Sloutsky and Fisher, 2012).

Although the debate surrounding the nature of the mechanism underlying the naming effect continues (Noles and Gelman, 2012; Sloutsky and Fisher, 2012), considerable evidence exists that challenges the claim that names function only as features of objects. First, as described earlier, auditory stimuli such as tones, melodies, or mechanical noises do not facilitate children’s categorization, despite their potential to increase similarity between perceived entities (Balaban and Waxman, 1997; Fulkerson and Waxman, 2007; Ferry et al., 2010). Second, by 16 months of age, infants are highly selective in the type of words they will use to guide their inductive inferences, taking into account the form class of the word and its role within language. For example, Keates and Graham (2008) demonstrated that 16-month-olds used novel words to license their inductive inferences only when those words were presented referentially (i.e., by a live speaker vs. a recorded instruction), embedded within an intentional naming phrase (vs. presented alone), and marked as count nouns (vs. adjectives). When these conditions were not met, infants disregarded the novel words and relied on perceptual similarity alone to guide their inferences about shared non-obvious properties. Furthermore, there is considerable developmental continuity in the link between count nouns, object categories, and category-based inductive inferences. For example, Graham et al. (2012) recently demonstrated that only consistently applied count nouns will guide 4-year-olds’ inductive inferences. In this experiment, two perceptually dissimilar category standards were labeled with count nouns, adjectives, or stickers. These markers were either applied consistently to, or varied across, the two standards. Results indicated that children formed an inclusive category only when the two standards were named with the same count noun. In all other conditions, children used shared perceptual similarity to guide their inferences.

In the present study, we focused on a developmental gap in the literature regarding infants’ attention to distinct labels. More specifically, we asked whether infants would recognize that distinct labels mark different categories, even if the label information conflicts with the perceptual similarity of objects. Research has demonstrated that when two objects are labeled with different count nouns, 13-month-old infants will rely on the perceptual similarity of the objects to guide their inductions (Graham et al., 2004). In this study, infants’ inferences patterned similarly to those of infants for whom the objects were not labeled, suggesting that infants essentially disregarded the different count nouns. In contrast, by 24 months of age, infants will privilege the count noun label over perceptual information (Jaswal, 2004; Jaswal and Markman, 2007). For example, when a cat-like animal was referred to as a dog, 24-month-olds inferred that this animal ate bones, rather than drank milk (Jaswal and Markman, 2007). In the present study, we examined the possibility that sensitivity to different labels as markers of distinct categories may become evident around the time that infants begin to show sophisticated attention to different types of labels in induction paradigms, namely in the 15- to 16-month range (Keates and Graham, 2008).

The goal of the present study was to examine whether 15-month-olds would inhibit their generalizations of target properties to test objects that were described with different labels, even if the labels conflicted with object appearance (i.e., target and test objects were highly similar). Using a general imitation paradigm, infants were presented with novel target objects that possessed non-obvious properties. The experimenter demonstrated the non-obvious property on the target object and observed whether or not the infant attempted to elicit the same property on test objects. Test trials consisted of objects that varied in shape similarity relative to the target objects (i.e., high and low-similarity). In the *Same Label* group, the experimenter introduced the target and test objects using the same novel label [e.g., “This is a *blick*.” (target) and “This is a *blick*.” (test)]. In the *Distinct Label* group, the experimenter labeled the target and test objects with different count nouns [e.g., “This is a *flum*.” (target) and “This is a *wug*. This is not a *flum*.” (test)].

Our predictions varied according to the label group to which infants were assigned. First, when target and test objects were described with the same count noun (i.e., Same Label group), we predicted that infants would generalize the non-obvious property to both high- and low-similarity test objects. This prediction followed from the finding that shared labels signal shared category membership (e.g., Keates and Graham, 2008). Second, when target and test objects were described with different count nouns (i.e., Distinct Label group), we expected infants to inhibit their generalizations of the properties to both high- and low-similarity test objects. This prediction followed from the finding that distinct labels do not foster categorization (e.g., Waxman and Braun, 2005), but rather highlight differences between objects (Xu, 1999).

## Materials and Methods

### Participants

Data from 36 15-month-old infants were included in the final sample. Infants were randomly assigned to one of two groups: the *Distinct Label* group (*n* = 18; *M* = 15.36 months; *SD* = 0.29; 9 males) or the *Same Label* group (*n* = 18; *M* = 15.61 months; *SD* = 0.26; 8 males). An additional 15 infants were tested but excluded due to excessive fussiness (*n* = 6), parental interference (*n* = 3), experimenter error (*n* = 1), or because their data were statistical outliers (*n* = 5; see Coding and Data Screening). Infants were from homes in which English was the primary spoken language, were from socioeconomic backgrounds that varied within the middle-class range (although this was not formally assessed), and were primarily Caucasian.

### Materials

**Figure 1:**
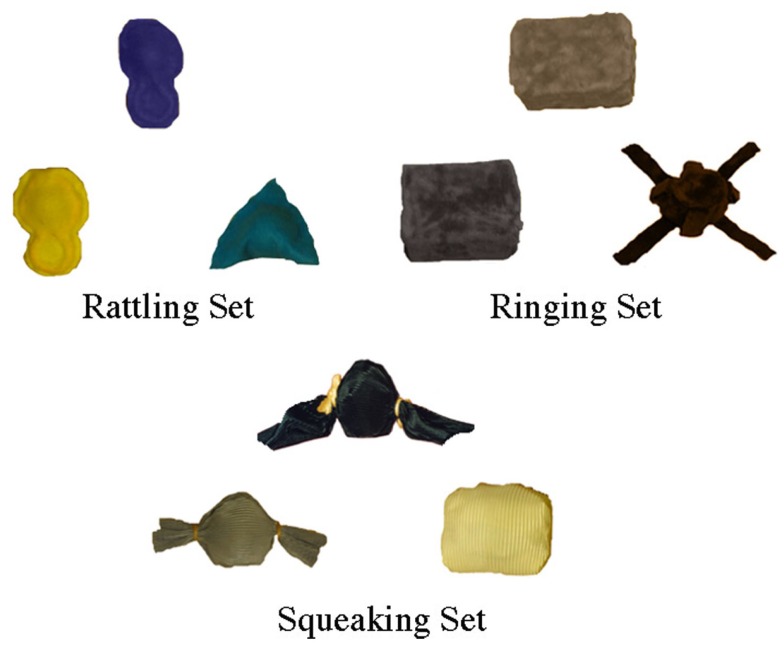
**The three object sets: the ringing set, the rattling set, and the squeaking set**. Within each set, the objects are arranged in the following manner: target object (top center), high-similarity test object (bottom left), and the low-similarity test object (bottom right).

Stimuli consisted of three object sets: a squeaking set, a ringing set, and a rattling set (see Figure 1 for object sets). For each set, a target object, a high-similarity test object, and a low-similarity test object were created. The high-similarity object was the same shape and texture as the target object, but differed in size and color. The low-similarity object was a different shape, size, and color than the target object, but shared the target object’s texture. All of the object sets possessed a non-obvious property (i.e., made a sound) that could be elicited by a specific action; when squeezed, the squeaking set squeaked, when tapped, the ringing set rang, and when shaken, the rattling set rattled. Two versions of each object set were created: a version for which the test objects possessed the non-obvious sound property and a version for which the test objects had been disabled such that they no longer possessed the non-obvious property (i.e., did not make a sound).

### Design

For each participant, one of the three object sets was presented in one of three within-subjects expectation conditions: the *unpredicted* condition, the *baseline* condition, and the *predicted* condition (see Table 1 for an overview). That is, for each infant, one object set was presented in the unpredicted condition, another set was presented in the baseline condition, and the third set was presented in the predicted condition. The condition of interest was the *unpredicted* condition, in which the target object had a non-obvious property, but the test object had been disabled. This condition was used as a measure of whether infants expected the target and test objects to share a non-obvious property. Specifically, if infants expected the test object to have the same non-obvious property as the target object, but were not able to elicit the sound because the test object had been disabled, they should persist in performing target actions on the test object. In the *baseline* condition, the target object and the test object did not possess the non-obvious property. Accordingly, this condition provided a baseline measure of infants’ exploratory actions. In the *predicted* condition, both the target object and the test object possessed the non-obvious property. This condition was used to maintain infants’ interest in the objects, so that they would not become bored or frustrated. The condition-object set pairings were counterbalanced across infants.

**Table 1:** **Overview of the three within-subject conditions**.

Condition	Infants’ expectation	Presence of the non-obvious property	
		Target object	Test objects
Baseline	None	Absent	Absent
Predicted	Fulfilled	Present	Present
Unpredicted	Violated	Present	Absent

*“Infants’ expectation” summarizes infants’ expectations about the test object in a given condition. Specifically, it refers to whether infants would expect the test object to have the non-obvious property and whether this expectation was met*.

Test trials were presented in two blocks. Each block consisted of three trials: one trial in the unpredicted condition, one in the predicted condition, and one in the baseline condition. For each trial, a target object and then a test object from the same set were presented. Objects from each set were presented in each block. For example, the target and high-similarity test objects from the ringing set might be presented in the first block, and the target and low-similarity test objects from the ringing set might be presented in the second block. The order of presentation of test objects within each block, as well as the order of presentation of conditions, was counterbalanced across infants. Testing protocols were yoked across groups.

### Procedure

Infants sat across a table from the experimenter, either in a booster seat or on their parent’s lap. Parents were instructed not to direct, prompt, or cue their infant in any way for the duration of the session. Parents were further instructed to re-place objects on the table, directly in front of their infant, should the infant drop an object off the table or pass an object to them.

Prior to the test trials, three warm-up trials were administered with the aim of demonstrating to the infants that they should imitate the experimenter’s actions when appropriate. For each of these trials, the experimenter demonstrated a target action on the warm-up object, and then handed the object to the parent. The parent performed the same action on the object, and then handed it to the infant so that he or she could imitate the demonstrated action. Regardless of whether they had imitated the target actions, all participants proceeded from the warm-up phase to the testing phase.

At the beginning of each test trial, the experimenter placed the target object on the table directly in front of the infant, out of his or her reach. She then introduced the object with a general attentional phrase (e.g., “Look. Look at this.”). In the predicted and unpredicted conditions, the experimenter then performed the target action that elicited the object’s non-obvious property. She did this action five times while drawing the infant’s attention to the object (e.g., “Look. See what this can do.”). In the baseline condition, no target action was performed, as the target objects did not possess a non-obvious property that could be demonstrated. The experimenter then proceeded to label the target object with a novel name (e.g., “Look. This is a *blick*”). For each target object, the novel name was repeated six times. Following this introduction, the infant was permitted to explore the object for 10 s, after which the experimenter retrieved the target object and placed it on the table within the infant’s view, but out of his or her reach.

The procedure then diverged according to the infant’s assigned group. In the Distinct Label group, the experimenter introduced the test object using a different novel name than that used to introduce the target, clearly emphasizing that the test object did not share the same name as the target object (e.g., “Look! This is a *wug*. Here is a *wug*… This is not a *blick*. No, this is not a *blick*.”). The new label was repeated six times across the introduction. In the Same Label group, the experimenter introduced the test object in the same way that she had introduced the target object – that is, using the same novel name. To ensure that the length of the introduction was similar to that for infants in the Distinct Label group, general attentional phrase were inserted between the labeling phrases (e.g., “Look! This is a *blick*. Here is a *blick*. …Look here! Yes, look here!”). If the object was dropped off the table or moved out of the infant’s reach, the experimenter (or parent) placed the object back in front of the infant within his or her reach. The same procedure of introducing the target object, allowing the infant to explore the target object, introducing the test object, and allowing the infant to explore the test object, was repeated for all trials.

### Coding and data screening

The number of target actions infants performed on the target and test objects was recorded by coders, based on a detailed scheme with specific criteria for the target action for each object set. The coders were unaware of the experimental hypotheses and group assignment, and could not distinguish the expectation conditions from each other on the basis of the videos. The target action for the squeaking set consisted of a squeezing motion performed with the hand. To be considered a target action, the infant’s fingers had to squeeze together on the object. Releasing the object did not count as a second action. If the infant squeezed the object with two hands, it was considered a single action, unless the two squeezes occurred independently. The target action for the ringing set consisted of a quick tapping or patting motion performed with the hand. To be considered a target action, the infant had to bring his or her hand down and make contact with the object. The upward motion performed as the infant raised his or her hand from the object following a target action was not counted as a second action. Likewise, touching the top of the object without performing a patting or tapping motion (e.g., to feel or poke it) was not considered a target action. If the infant tapped the object with two hands, it was counted as one action, unless the taps occurred independently. The target action for the rattling set consisted of a back and forth, upward, or downward motion performed with the object in hand. The action could be performed with the infant’s wrist and/or entire arm. A fluid back and forth or up and down motion (i.e., shaking the object in one direction, followed by the rebound from that motion) was considered one target action, but if there was a pause between the two motions (i.e., shaking the object in one direction, then shaking the object in another direction) they were considered two actions. Squeezing or tapping the object, hitting a body part or the table with the object, or manipulating the object in order to visually examine it or to throw/pass it to the parent or experimenter, was not counted as a target action. If the infant shook the object with two hands together, it was counted as only one action.

Twenty percent of the data (*n* = 7) was re-coded by a second person to obtain a measure of interrater reliability. Intraclass coefficients for target and test object frequency ratings were 0.998 (*p*s < 0.001).

Infants whose standard scores for frequency of the target actions were more than 3.0 standard deviations above or below the mean in the unpredicted or baseline condition were considered statistical outliers and were removed from the data analyses (*n* = 5).

## Results

Infants’ responses on the warm-up trials did not vary significantly across label groups (*p* > 0.31). Similarly, 18/18 infants in the Same Label group and 16/18 infants in the Distinct Label groups performed at least one target action on the target objects in the unpredicted and predicted conditions (recall that no action was demonstrated in the baseline condition). These analyses indicate that infants in both groups were willing to imitate the experimenter’s actions on objects.

**Figure 2:**
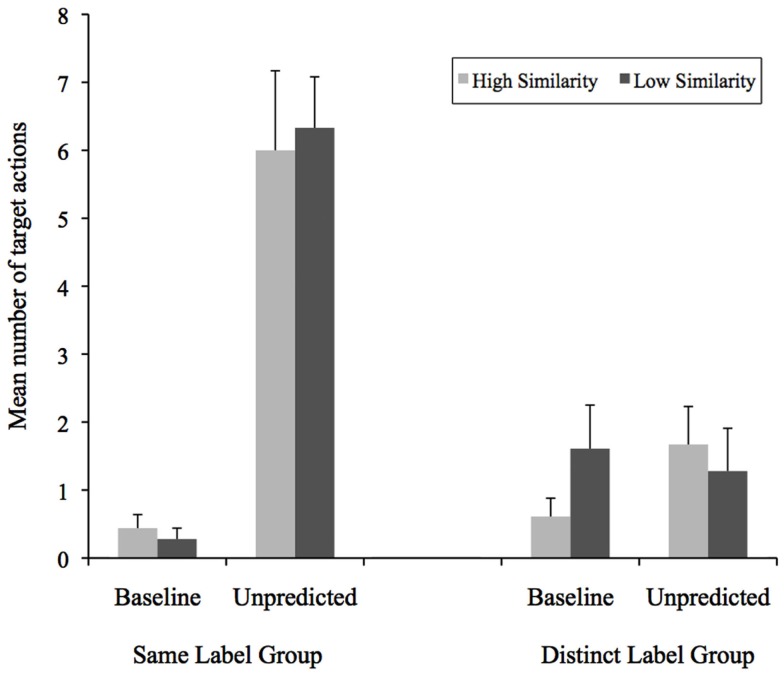
**Mean number of target actions performed on test objects as a function of label group, condition, and similarity**.

Our primary analyses focus on infants’ performance of actions on the test objects in the unpredicted condition and baseline condition. See Figure 2 for the mean number of target actions performed as a function of similarity, condition, and label group. We did not analyze the data from the predicted condition, as it was difficult to interpret why infants continued to perform target actions on test objects. Specifically, it was not possible to distinguish between target actions performed because infants expected the test object to have the same non-obvious property as the target object, and target actions performed as a result of the reinforcing nature of the sound property elicited by previous target actions (for further discussion of this problem see Baldwin et al., 1993; Welder and Graham, 2001; Graham et al., 2004).

To examine whether infants’ performance of target actions on the test objects varied as a function of condition, shape similarity to the target, and consistency of the label presented, we conducted a 2 (Label Group) × 2 (Condition) × 2 (Shape Similarity) mixed factor ANOVA. This analysis yielded a significant main effect of label group, with infants in the Same Label group (*M* = 3.26, SD = 1.80) performing more target actions than infants in the Distinct Label group, (*M* = 1.29, SD = 1.28), *F*(1, 34) = 14.30, $\eta_{p}^{2}=0.30,$ *p* < 0.001. There was also a main effect of condition with infants performing more target actions in unpredicted condition (*M* = 3.81, SD = 3.66) than in baseline condition, (*M* = 0.74, SD = 1.23), *F*(1, 34) = 37.66, $\eta_{p}^{2}=0.53,$ *p* < 0.001. The interaction of label group by condition, *F*(1, 34) = 29.36, $\eta_{p}^{2}=0.46,$ *p* < 0.001, was also significant. There was no significant main effect, nor any significant interactions, involving shape similarity (*p*s > 0.15).

We followed up on the group by condition interaction in two sets of analyses. Recall that the baseline condition was a control condition included to ensure that the appearances of the objects did not suggest the target actions. Thus, we expected that infants would rarely, if ever perform target actions on the target objects and we did not expect the target actions to vary by label group or shape within this condition. First, we examined infants’ expectations about shared properties by comparing target actions infants performed on test objects in the unpredicted condition (where they had seen a functional target object) versus the baseline condition (where they had seen a non-functional target object) as a function of label group (collapsed across shape similarity). If infants expect the target and test objects to share a property, then they should perform more actions in the unpredicted condition than in the baseline condition. If they did not expect the target and test object to share a property, then there should be no difference between the baseline and unpredicted conditions. In the Same Label group, infants performed significantly more target actions on the test objects in the unpredicted condition (*M* = 6.62, SD = 3.40) than in the baseline condition (*M* = 0.36, SD = 0.58), *t*(17) = 7.45, *p* < 0.0001. In the Distinct Label group, however, infants did not differ significantly in number of target actions performed in the unpredicted condition (*M* = 1.47, SD = 2.09) versus the baseline condition (*M* = 1.11, SD = 1.59), *t*(17) = 0.57, *p* > 0.57.

Next, we assessed whether the number of actions performed on objects varied as a function of label group in the baseline and unpredicted conditions separately. As expected, a 2 (Label Group) × 2 (Shape Similarity) ANOVA yielded no significant main effects or interactions (*p*s > 0.07) for the baseline condition. Recall that we expected that the type of label would influence infants’ performance of target actions in the unpredicted condition. In particular, we predicted that when target and test objects were labeled with a consistent name, infants would generalize object properties to both the high- and low-similarity object, based on previous research (e.g., Welder and Graham, 2001; Keates and Graham, 2008). When the target and test objects were labeled with distinct names, we predicted that infants would inhibit their generalization of the properties and be less likely to generalize the properties to either the high- or low-similarity object. These predictions were confirmed by a 2 (Label Group) × 2 (Shape Similarity) ANOVA, which yielded only a significant main effect of label group, *F*(1, 34) = 24.77, $\eta_{p}^{2}=0.42,$ *p* < 0.001. That is, infants in the Same Label group performed significantly more target actions on the test objects (*M* = 6.17, SD = 1.47) than infants in the Distinct Label group (*M* = 1.47, SD = 2.10), collapsed across shape similarity.

The results of the above analyses indicate that naming target and test objects with the same name led infants to infer that both high- and low-similarity test objects shared the non-obvious property of the target. In contrast, distinct names significantly reduced infants’ performance of target actions relative to the Same Label group. In fact, distinct names reduced infants’ performance of target actions in the unpredicted condition to the level of the baseline condition (where no actions had been demonstrated). In the next analysis, we directly assessed whether this difference held for the high shape similarity test object in particular. Results of a planned comparison indicated that infants in the Same Label group performed significantly more actions on the high-similarity test object than infants in the Distinct Label group, *p* < 0.01.

The results from the Distinct Label group raise the possibility that distinct labels are overriding infants’ reliance on shape. Previous research has demonstrated that infants and preschoolers tend to attend to shape to guide their inductions, in the absence of shared count noun labels (e.g., Welder and Graham, 2001; Keates and Graham, 2008; Graham et al., 2012). In particular, results of research using a similar paradigm to that used in the current study has demonstrated that infants aged 13 months (Graham et al., 2004), 14 months (Graham and Kilbreath, 2007), 15 months (Graham and Diesendruck, 2010), 16 months (Keates and Graham, 2008), 18 months (Welder and Graham, 2001), and 22 months (Graham and Kilbreath, 2007) show strong attention to shape when no label is provided. This attention to shape has also prevailed when objects have been given labels that infants do not view as marking shared kind (i.e., adjectives; Keates and Graham, 2008).

In the next set of analyses, we directly examined whether naming the target and test objects with a distinct label reduced infants’ reliance on shape to guide their inductions, relative to a no label group. Given that numerous studies with infants both younger and older than the infants tested in the current experiment have demonstrated that infants rely on shape to guide their inductions in no label groups, we drew upon an existing data set to make these comparisons. That is, we compared infants’ performance in the Same Label and Distinct Label groups to that of 16-month-old infants in a No Label group (unpredicted condition), from a previously published experiment (Keates and Graham, 2008). The infants in the latter group were recruited in a similar fashion and tested using the same stimuli and experimental methods as the infants in the present study. In this group, the target and test objects were introduced using general attentional phrases (e.g., “Look at this one”). Although this group of infants is, on average, 1 month older than the infants in the present study, previous research has demonstrated developmental consistency in infants’ reliance on shape to generalize novel properties in the absence of shared labels, in infants ranging in age from 13- to 22-months of age (including 15-month-old infants).

**Figure 3:**
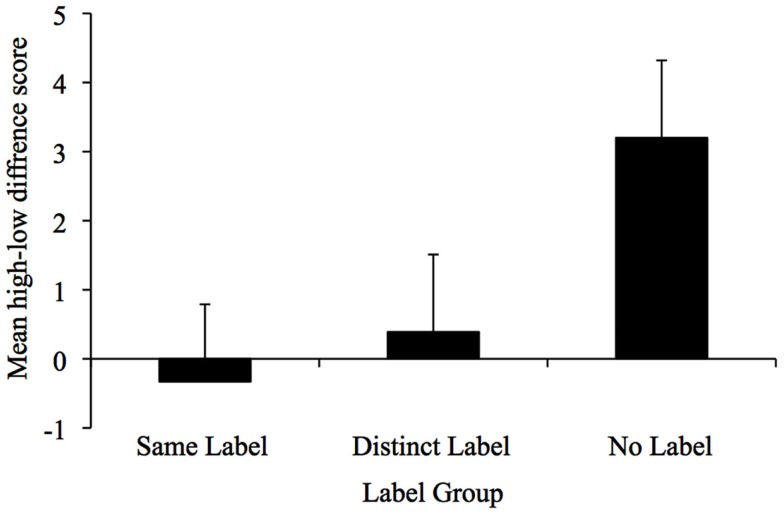
**Mean high-low difference scores as a function of label group**.

To conduct comparisons between the No Label data and the data from the present study, we first calculated a shape reliance difference score that captured the degree to which infants’ privileged shape in their inductive inferences. This score was calculated by subtracting the number of target actions performed on the low-similarity test object from those performed on the high-similarity object. We chose to use a difference score, rather than number of target actions to take into consideration individual differences in interest level, motivation, and attention toward the objects, as well as the 1 month age difference between infants in the No Label group and the two label groups tested in this study. Thus, the difference score enabled us to assess relative changes in each infant’s actions on the high- and low-similarity test objects. Difference scores in the positive range would indicate relatively greater reliance on shape. See Figure 3 for difference scores as a function of label group. Results of a one-way ANOVA indicated a significant main effect of group, *F*(2, 53) = 3.54, $\eta_{p}^{2}=0.12,$ *p* < 0.05. Planned comparisons indicated that shape reliance difference scores did not vary reliably between infants in the Distinct Label group (*M* = 0.39, SD = 2.83) and the Same Label group (*M* = −0.33, SD = 4.79), *p* > 0.59. Both groups did, however, differ significantly from infants in the No Label group (*M* = 3.20, SD = 2.83), *t*(36) = 2.09, *p* < 0.05 and *t*(36) = 2.12, *p* < 0.05. Inspection of the difference scores demonstrate that only infants in the No Label group showed a preference to extend properties based on shape.

## Discussion

The present study examined the role of common and distinct labels in guiding 15- to 16-month-old infants’ inductive inferences. When target and test objects were labeled with the same novel count noun, we expected that infants would infer that objects shared non-obvious properties, regardless of shape similarity. In contrast, when target and test objects were labeled with distinct count nouns, we expected that infants would be less likely to infer that objects shared non-obvious properties, even if they were highly similar. The results of the present study support our initial predictions and offer insight into the conditions under which infants will rely on object labels to generalize non-obvious properties.

Our results reinforce previous findings that count noun labels license infants’ inductive inferences. When objects were labeled with the same count noun, infants generalized the non-obvious property to both high- and low-similarity objects, suggesting that they formed an inclusive category despite varying degrees of perceptual similarity. This finding is consistent with previous research demonstrating that the effect of perceptual similarity is diminished or disregarded when objects are labeled with the same count noun (e.g., Graham et al., 2004; Graham and Kilbreath, 2007; Keates and Graham, 2008). When target and test objects were labeled with distinct count nouns, infants were significantly less willing to generalize the non-obvious property to test objects, regardless of perceptual similarity. In this case, distinct labels led infants to restrict their generalizations of the non-obvious property, suggesting that they had carved the perceptually similar objects into distinct categories. Critically, infants relied on information about object kind, as marked by count noun labels, rather than perceptual similarity to guide their inferences.

Our findings fill a developmental gap in the literature regarding infants’ attention to distinct labels. As discussed earlier, research using the same paradigm has demonstrated that when target and test objects were introduced with different labels, 13-month-old infants continued to generalize properties to high-similarity objects (Graham et al., 2004). The present results suggest that between 13- and 15-months, there is a developmental shift in the degree to which infants rely on high shape similarity as an indicator of category membership when faced with contradictory information. Specifically, infants’ reliance on perceptual information to guide their inferences, when labels contradict this information, decreases and as a result, infants begin to inhibit their generalizations to highly similar objects that have been labeled with distinct nouns. What might account for this shift? We suggest, speculatively, that greater word knowledge, as well as increased inhibitory control, may lead infants to inhibit their generalization of target properties to differently labeled objects despite contradictory perceptual information. Indeed, when tested in tasks with minimal motor requirements (i.e., looking-time paradigms) infants as young as 10-months-old expect different labels to refer to different kinds, regardless of perceptual similarity (e.g., Dewar and Xu, 2009).

Our findings dispute the proposal that nouns act as additional features of objects, contributing to overall similarity. Recall that proponents of this perspective propose that entities that share labels are perceived as more similar (e.g., Sloutsky and Fisher, 2004). In the present study, if labels were acting as features, we would have expected to find a greater number of inferences to the high-similarity test objects compared to the low-similarity objects in the Same Label group, as similarity computations would yield an additive effect of label and shape. In other words, the overall similarity computation would be higher for high-similarity than low-similarity test objects. Our results, however, did not support this account; infants did not generalize the non-obvious property to the high-similarity object more than to the low-similarity object when they were named with the same count noun. Furthermore, although the phenomenon of auditory overshadowing (i.e., greater salience of auditory information over visual information when the two are presented simultaneously; Sloutsky and Napolitano, 2003), might predict that shared labels would be more heavily weighted than shape similarity, one would not expect latter to be disregarded completely. That is, overall similarity (composed of both linguistic labels and perceptual information) should continue to be computed despite auditory overshadowing. Accordingly, the overall similarity computation would continue to be higher for high-similarity than low-similarity test objects, which should have resulted greater generalization to the high-similarity than the low-similarity object. As noted above, this prediction was not borne out by our findings. Furthermore, given that shape similarity is always a shared feature of the target and high-similarity test objects, similarity-based accounts would predict that distinct labels should inhibit generalization to a greater extent for low-similarity objects than for high-similarity objects. That is, the overall similarity computation would be lower for low-similarity than high-similarity objects. Again, our results do not support this account; infants’ generalization of the non-obvious property (or lack thereof) did not differ significantly for the high-similarity object or the low-similarity object in the Distinct Label group.

In summary, the present findings have advanced our understanding of infants’ inductive abilities, demonstrating that by 15 months of age, infants have a sophisticated understanding of the role of count noun labels in guiding inductive inferences. That is, our findings indicate that 15-month-old infants appreciate that shared count noun labels license inductive inferences, consistent with the expectation that shared count noun labels index shared category membership and shared category membership promotes inductive inferences. Furthermore, our findings provide the first evidence that naming objects with distinct labels leads 15-month-old infants to inhibit their generalization of properties, even when these objects are highly perceptually similar. This suggests that by 15-months of age, infants appreciate that distinct count noun labels mark distinct categories of objects, even for cases in which objects are highly perceptually similar.

## Conflict of Interest Statement

The authors declare that the research was conducted in the absence of any commercial or financial relationships that could be construed as a potential conflict of interest.
